# iMITEdb: the genome-wide landscape of miniature inverted-repeat transposable elements in insects

**DOI:** 10.1093/database/baw148

**Published:** 2016-12-26

**Authors:** Min-Jin Han, Qiu-Zhong Zhou, Hua-Hao Zhang, Xiaoling Tong, Cheng Lu, Ze Zhang, Fangyin Dai

**Affiliations:** 1 State Key Laboratory of Silkworm Genome Biology, Key Laboratory for Sericulture Functional Genomics and Biotechnology of Agricultural Ministry, Southwest University, Chongqing 400715, China; 2 Laboratory of Evolutionary and Functional Genomics, School of Life Sciences, Chongqing University, Chongqing 401331, China; 3 College of Pharmacy and Life Science, Jiujiang University, Jiujiang 332000, China

## Abstract

Miniature inverted-repeat transposable elements (MITEs) have attracted much attention due to their widespread occurrence and high copy numbers in eukaryotic genomes. However, the systematic knowledge about MITEs in insects and other animals is still lacking. In this study, we identified 6012 MITE families from 98 insect species genomes. Comparison of these MITEs with known MITEs in the NCBI non-redundant database and Repbase showed that 5701(∼95%) of 6012 MITE families are novel. The abundance of MITEs varies drastically among different insect species, and significantly correlates with genome size. In general, larger genomes contain more MITEs than small genomes. Furthermore, all identified MITEs were included in a newly constructed database (iMITEdb) (http://gene.cqu.edu.cn/iMITEdb/), which has functions such as browse, search, BLAST and download. Overall, our results not only provide insight on insect MITEs but will also improve assembly and annotation of insect genomes. More importantly, the results presented in this study will promote studies of MITEs function, evolution and application in insects.

**Database URL:** http://gene.cqu.edu.cn/iMITEdb/

## Introduction

Miniature inverted-repeat transposable elements (MITEs) were first discovered in plants, and are widely distributed in eukaryotes (1–5). MITEs belong to class II (or DNA) transposable elements (TEs), and are non-autonomous elements derived from the internal-deletion of autonomous DNA transposons (6, 7). However, they can be mobilized by transposases encoded by their parental autonomous transposons (called trans-mobilization) or non-parental elements (called cross-mobilization) (8, 9). MITEs can be classified into different superfamilies based on the nucleotide composition of terminal inverted repeats (TIRs) and target site duplications (TSDs). Unlike other DNA transposons, MITEs often have some obvious characteristics: shorter sequence length (<800 bp), high AT content, insertion preference in or near genes and high copy numbers in a genome (10–12).

MITEs have attracted widespread attention due to their roles in gene expression, genome evolution and phenotypic diversity (13–16). MITEs not only up-regulate the expression of nearby genes by acting as new cis-regulatory elements but also down-regulate or silence the expression of some genes by small RNAs derived from these elements at the transcriptional and/or post-transcriptional levels (14, 17–20). Besides, MITEs make a great contribution on the evolution of genome size (12, 16). Furthermore, MITEs are considered as a good genetic source applied in DNA makers, transgenic vectors and effective insertion mutagen (21–24). However, most of above results were obtained from studies of plants.

Since more and more genome sequences become available, several computer programs have been developed to identify MITEs in genomes, and a larger number of MITEs have been identified in the eukaryotic genomes especially in plant genomes (13, 20, 25–28). Although several studies tried to identify MITEs in the insect genomes (3, 5, 29), the number of reported MITEs could be just the tip of the iceberg with rapidly increasing insect genome were released.

In the present study, MITEs from 98 insect genomes were identified, classified and annotated using MITE-Hunter and Repetitive Sequence with Precise Boundaries (RSPB) as well as a series of Perl scripts. We identified 6012 MITE families belonging to 16 known superfamilies in these genomes. In total 5701 of 6012 MITEs families are novel and have no matches to the previously known MITEs in the databases of Repbase and NCBI non-redundant nucleotide database. The abundance of MITEs varies greatly among the different insect species and significantly correlated with genome size. Finally, all identified MITEs are made available in a newly constructed database called iMITEdb.

## Materials and methods

### Data sources used in this study

Ninety-eight released insect genomes including Coleoptera (7 species), Diptera (48 species), Hemiptera (8 species), Hymenoptera (20 species), Lepidoptera (9 species), Strepsiptera (1 species), Orthoptera (1 species), Odonata (1 species), Isoptera (1 species), Thysanoptera (1 species) and Ephemeroptera (1 species) were downloaded from NCBI (http://www.ncbi.nlm.nih.gov/) (as of 8 March 2015) (Supplementary Table S1).

### Identification, classification and characterization of insect MITEs

MITE-Hunter and RSPB were used to search for MITEs in 98 insect genomes (20, 27). Briefly, the pipeline for MITEs identification included four steps (Supplementary Figure S1): (i) First, MITE-Hunter was used to search insect genomes for candidate MITEs. Then, RSPB was used to identify potential insect MITEs. In RSPB, the hunter2ref.pl script, a Perl script of RSPB, was used to skip the confirmed MITEs identified by MITE-Hunter; (ii) Each candidate MITE was used as a query in BLASTN (*e*-value < *e*^−^^6^) search against the corresponding genome sequence. Candidate MITE families with copy numbers <3 were discarded. Then, multiple sequences retrieved by each candidate MITE were aligned using MUSCLE (30); (iii) Consensus sequence was generated using a Perl script, and consensus sequences >800 bp in length were discarded; (iv) Finally, the TSDs and TIRs of each MITEs were retrieved using Perl script. MITEs from each species were assigned into families through all-versus-all BLAST method. The same family was defined by nucleotide identity>80%, BLAST *e*-value < *e*^−^^6^ and percent query coverage >80%. MITEs were classified into superfamily based on TIRs and TSDs (16).

### Construction of insect MITEs database

A database containing the information of all insect MITEs identified in this study was constructed using Linux, PHP, Apache, MySQL and Perl as well as Common Gateway Interface.

## Results and discussion

### Identification, classification and abundance of MITEs in 98 insect genomes

In this study, a total of 6012 MITE families were identified in 98 insect genomes. The consensus sequences of these MITE families were used as queries in BLASTN (*e*-value < 10^−^^10^) searches against the Repbase and NCBI non-redundant nucleotide database. Both databases include almost all known MITEs. We found that 5701 (∼95%) MITE families did not match to any known TEs in both databases. Therefore, these families were defined as novel MITE families. MITEs like other TEs are huge challenges for host genome sequencing, assembly and annotation due to their repeatability. Thus, the larger number of novel MITE families identified in this study will greatly improve sequencing, assembly and annotation of insect genomes, and facilitate the evolutionary and functional studies of MITEs in the future.

**Figure 1.**
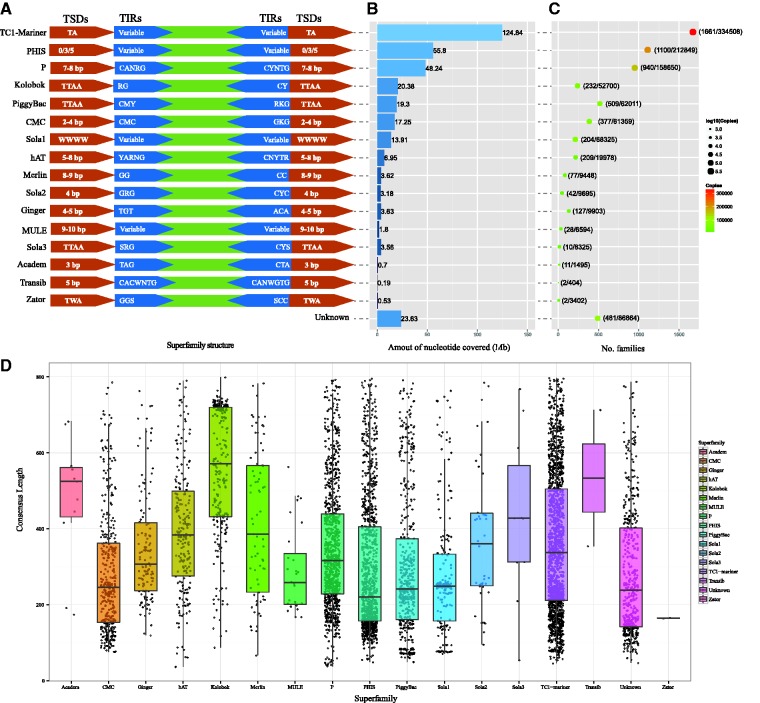
Characteristics of each MITEs superfamily in insect genomes. **(A)** Structure of each superfamily. TSDs sequence and TIRs are shown. **(B)** Amount of nucleotide covered of each superfamily in 98 insect genomes. **(C)** The number of families and copies of each superfamily in the investigated insect genomes. Numbers in parenthesis represents ‘families/copies’. **(D)** The distribution of consensus sequence length for each MITE superfamily.

Based on the characteristics of TSDs and TIRs, 5531 MITE families were classified into 16 known superfamilies including *TC1-Mariner*, *PHIS*, *P*, *Kolobok*, *PiggyBac*, *CMC*, *Sola1*, *hAT*, *Merlin*, *Sola2*, *Ginger*, *MULE*, *Sola3*, *Academ*, *Transib* and *Zator*. Meanwhile, 481 families could not be readily assigned to any known DNA transposon superfamilies, and were designated as unknown (Figure 1A). The abundance of different MITE superfamilies in the 98 insect genomes varies markedly. The largest MITE superfamily is the *TC1-Mariner* constituting of 124.84 megabase (Mb) in the insect genomes studied. The smallest superfamily is *Transib* cover 0.19 Mb (Figure 1B). The numbers of families and copies also vary greatly among the different MITE superfamilies. The number of families ranges from 2 to 1,661 and the number of copies ranges from 404 to 334, 508. *TC1-Mariner* is also the largest superfamily with high numbers of families and copies (Figure 1C). However, the average sequence length of *TC1-Mariner* (∼373 bp) superfamily is smaller than *Academ* (∼470 bp), *Kolobok* (∼386 bp), *Transib* (∼482 bp), *Sola3* (∼428 bp) and *Merlin* (∼383 bp) superfamilies (Figure 1D).

Such abundance of the *TC1-Mariner* superfamily in the insect genomes could be in part explained by its short TSDs because short TSDs likely have much more target sites in host genomes. *TC1-Mariner* transposons are prevalent in eukaryotes, and feature di-nucleotide (5′-TA-3′) TSDs (31). *TC1-Mariner* TSDs are the shortest among known DNA transposon superfamilies (*PiggyBac* is characterized by 5′-TTAA-3′ TSDs, *P* is 7-8 bp TSDs, *Academ* is 3 bp TSDs etc.) (32). TSDs of *Academ* are shorter than *PiggyBac*, *hAT*, *Merlin* etc. However, among the superfamilies, *Academ* has the lowest abundance. Thus, the number of target sites can not completely explain the abundance variation of transposon superfamilies.

The result of correlation analysis revealed that the abundance of each superfamily in insect genomes significantly correlated with the numbers of its families and copies (Supplementary Figure S2A and S2B), and have no significant correlation to its length (Supplementary Figure S2C). In general, the numbers of families and copies of a transposon superfamily are affected by their transposition activities, removal rate or host TE regulation and so on. Whether *TC1-Mariner* in insect genomes has higher transposition activity is to be experimentally verified in the future. If this is case, *TC1-Mariner* could be exploited as a good vector in insect transgenic technology.

### MITE abundance in 98 insect genomes

**Figure 2.**
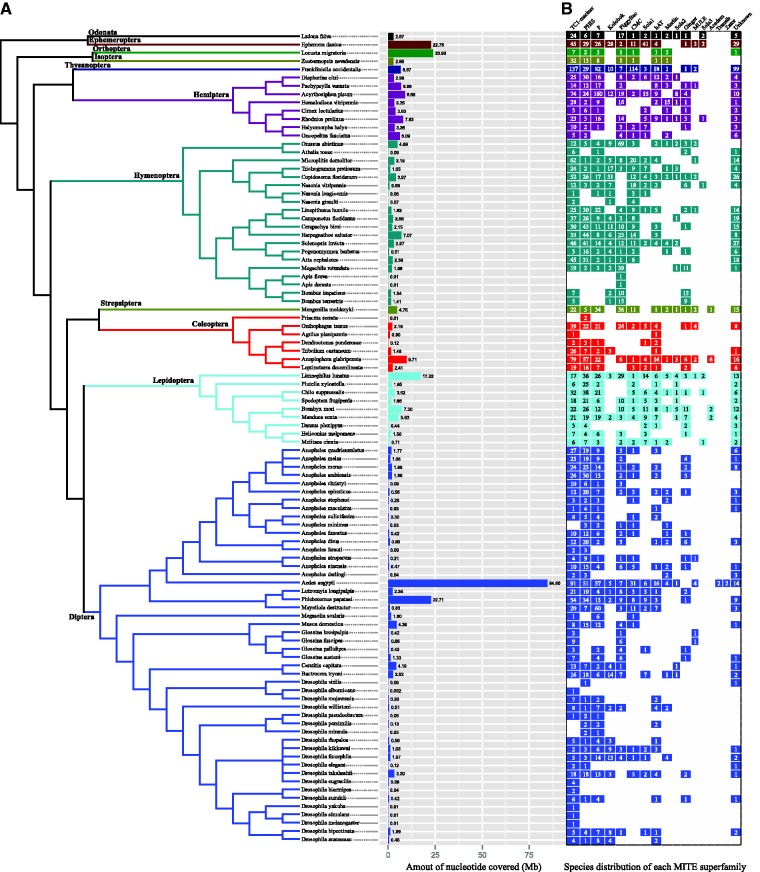
Distribution and abundance of MITEs in 98 insect genomes. **(A)** Amount of nucleotide covered of MITEs in each insect genome. Same color bars represent the same insect order. Numbers represent MITEs abundance (in megabase) in different insect genomes. **(B)** Distribution of MITEs superfamily in each insect genome, color boxes indicated presence; numbers within the color box represent the number of families.

To estimate the abundance of MITEs in each insect genome, the consensus sequence of each MITE family was used as query in a BLASTN search against corresponding genome. The results show that the abundance of MITEs varies greatly among the different genomes (Figure 2A). For instance, MITEs constitute ∼84.60 Mb (occupied ∼6.4% of the genome sequence) in the *Aedes aegypti* genome. But *Anopheles darlingii* harbors only ∼0.04 Mb (∼0.03% of genome) MITE sequences in its genome. Similarly, there are ∼2.18 Mb MITE sequences in the *Onthophagus taurus* genome, whereas only ∼0.9 Mb in the *Agrilus planipennis* genome. In addition, the numbers of MITE superfamilies and families also vary markedly among the different genomes (Figure 2B). For example, 80 families with 7 known superfamilies were detected in the *Megachile rotundata* genome, whereas only one family was identified in the *Apis florea* genome.

**Figure 3.**
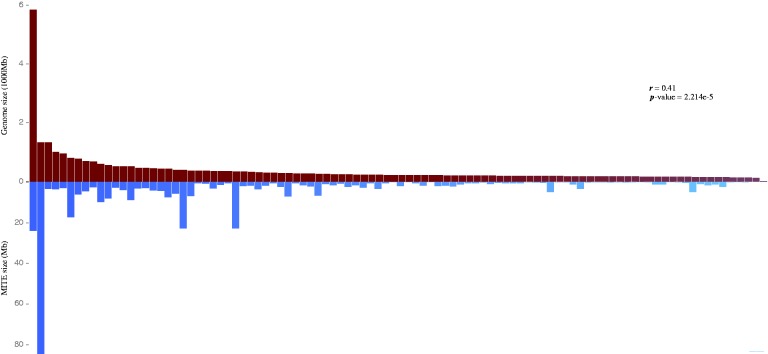
Correlation between the abundance of MITEs and genome size. Histogram above the graph (in red) represents distribution of genome size (unit—1000 megabase). Histogram below the graph (in blue) represents the distribution of MITEs abundance (unit—megabase). Correlation analysis was performed using the R program with the Pearson’s method.

We performed a correlation analysis between the abundance of MITEs in a species and the corresponding genome size and found a very significant correlation between the two characteristics (*r* = 0.41, *P*-value = 2.214e-5), indicating that the abundance of MITEs influences the insect genome size (Figure 3). This is consistent with the observation in plant genomes (16). When compared with other TEs, MITEs are shorter in a given genome. However, MITEs usually have high copy numbers (12). Thus, they may have an important role in the evolution of the genome size.

### Construction of insect MITEs database

**Figure 4.**
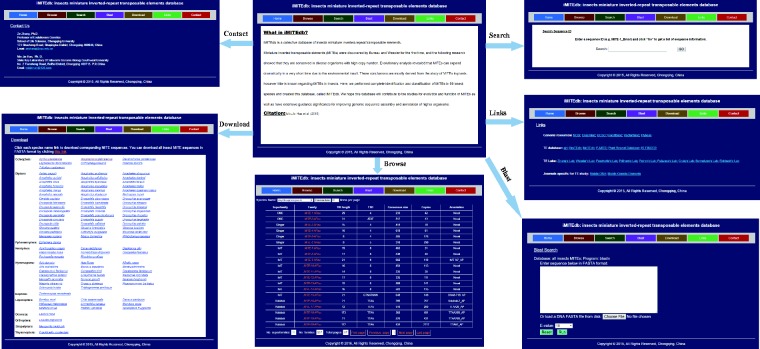
The web interface of iMITEdb. The interfaces had browse, search, blast, download, links and contacts.

We constructed a database, called iMITEdb, using the MITE sequences identified in this study and other available MITE information. The iMITEdb contains the following functions: browse, search, BLAST and download (http://gene.cqu.edu.cn/iMITEdb/) (Figure 4). Each MITE family in the database includes species, superfamily name, family name, TIRs, TSDs, copies, length and consensus sequence. This database allows browsing the information of interesting MITEs based on insect species, superfamily and family. Users can also perform individual search based on a MITE name to obtain information about each MITE family. In BLAST searches, users can enter a sequence in FASTA format or load a DNA sequence containing file to perform BLASTN search against all identified MITEs. All MITE sequences available in the database can also be downloaded. This will greatly facilitate the studies of function and evolution of MITEs in insects in future.
